# Assessment of clinician agreement with and knowledge of evidence‐based obesity treatment in the primary care setting

**DOI:** 10.1002/osp4.70011

**Published:** 2024-10-01

**Authors:** Angela R. Baalmann, Melissa C. Norton, Natalie R. Gadbois‐Mincks, Samuel Ofei‐Dodoo, Erica N. Presnell

**Affiliations:** ^1^ Fred Wilson School of Pharmacy High Point North Carolina USA; ^2^ Ascension Via Christi Hospitals Wichita Kansas USA; ^3^ Drake University College of Pharmacy and Health Sciences Des Moines Iowa USA; ^4^ The University of Kansas School of Medicine Wichita Kansas USA

**Keywords:** evidence‐based medicine, obesity, primary health care

## Abstract

**Introduction:**

Effective, evidence‐based obesity treatment is needed, which often involves use of anti‐obesity medications (AOMs). Data on the breadth and quality of guideline‐directed obesity treatment implementation in primary care remain limited. This study aimed to assess primary care clinicians' agreement with and knowledge of guideline‐directed obesity treatment, as well as to assess the health status of persons with obesity and their use of AOMs.

**Methods:**

This multimethod study included a prospective survey of primary care clinicians, utilizing a questionnaire that measured agreement on a 5‐point Likert scale and knowledge via multiple choice questions. A retrospective analysis was also performed of patient data collected between 30 June 2016 and 30 June 2020 from primary care clinics in the Midwest.

**Results:**

Data were analyzed from 27 clinician survey responders, finding agreement toward all measured areas, however less strong for chronic AOM use and resource allocation. The survey identified multiple gaps in knowledge. Researchers assessed 5656 baseline encounters and 2941 corresponding follow‐up encounters. Analysis revealed ≥50% of the total patients experienced persistently uncontrolled obesity (mean body mass index of ≥40 kg/m^2^) and weight‐related complications. Low rates (≤10%) of AOM use in clinically eligible patients were shown, with phentermine monotherapy being the most commonly used.

**Conclusions:**

Clinicians agree with guideline‐directed obesity treatment. Persons with obesity who are poorly controlled identify an opportunity for patient care improvement.

## Graphical abstract

Given that guideline‐directed obesity treatment is imperative in the primary care setting, this multimethod study aimed to assess its presence via a prospective clinician survey and retrospective patient data analysis. Twenty seven primary care clinicians completed the survey, reporting general agreement with guideline‐directed obesity treatment and demonstrating gaps in knowledge of evidence‐based practices. >2900 baseline and follow‐up patient care encounters were analyzed, revealing a lack of positive clinical outcomes and appropriate anti‐obesity medication prescribing patterns.

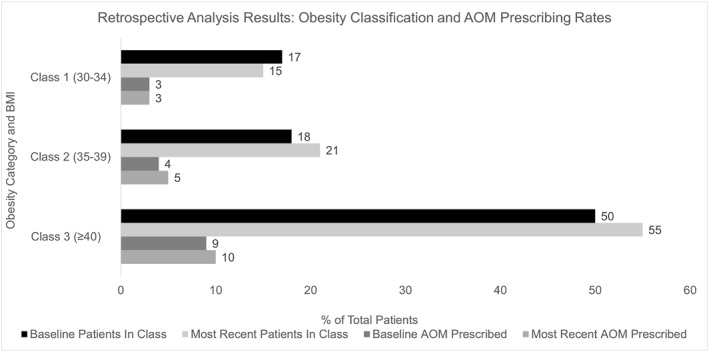

## 1 INTRODUCTION

Obesity is a chronic disease caused by an excess accumulation of adipose tissue and/or a dysregulation in its distribution or function.^1^ Each aspect of human physiology is affected by obesity, manifesting in a variety of weight‐related complications such as hypertension, type 2 diabetes mellitus, osteoarthritis, etc. Obesity causes significant physical, emotional, and psychological burdens for those diagnosed, and financial burdens for the US healthcare system as a whole.^2^ Currently, it appears that the majority of clinicians consider obesity a serious condition, whereas past stigma around obesity may have limited effective treatment.^3^, ^4^, ^5^ While perceptions of clinicians appear to be evolving to recognize the chronic, biological changes that occur in persons with obesity, it is unknown whether clinicians agree with use of proposed evidence‐based practices and anti‐obesity medications (AOMs), as described in clinical practice guidelines for obesity.

Interprofessional collaboration in the primary care setting may provide solutions to the rising number of persons with obesity, projected clinician shortages in this setting, and possibly improve the overall care of persons with obesity.^6^, ^7^ Team‐based care can provide needed personnel, and a diversity of skill sets and views, both of which increase the likelihood of improved patient outcomes. Involvement of dieticians, exercise physiologists, behavioral specialists, and pharmacists have all demonstrated benefit in improving outcomes related to obesity and its associated complications.^8^, ^9^, ^10^, ^11^, ^12^ Currently, studies of obesity often focus on patient perceptions and beliefs about the nature and impacts of the disease, or how these types of perceptions differ from that of their clinicians, rather than optimal, evidence‐based treatment of the disease.^3^, ^4^, ^5^, ^13^, ^14^, ^15^, ^16^, ^17^ Additionally, past studies focusing on clinician perceptions occurred prior to recent updates in obesity treatment strategies or did not assess clinician perceptions toward interprofessional collaboration.^13^, ^17^

This study focused on evaluating clinician agreement with and knowledge of guideline‐directed obesity treatment, while also analyzing real‐world patient data to highlight strengths and weaknesses in the care of persons with obesity. The primary outcome is to describe primary care clinician agreement with and knowledge of guideline‐directed obesity treatment. Secondary outcomes are to analyze demographics, clinical variables, and patterns of care in a select population of treatment eligible persons with obesity in the primary care setting. A connection may exist between agreement and perceptions. This evaluation will add to existing studies in this area. The results of this study are intended to inform clinicians of how care in a limited population of persons with obesity is being perceived and conducted. Such information may improve their practice and care of persons with obesity.

## 2 METHODS

### 2.1 Prospective clinician survey

Study investigators developed a questionnaire designed to meet study aims using a multi‐stage process to confirm its validity. A variety of question types were utilized to assess obesity treatment as delivered in primary care settings. Clinician agreement was assessed through 12 questions and measured on a 5‐point Likert scale. Questions focused on topics such as the nature of obesity as a disease, use of medications to achieve weight loss, and resources available in this setting for obesity treatment. Knowledge was assessed in seven questions by identifying correct responses to multiple choice questions. Question items were based on the guidelines from the American Association of Clinical Endocrinologists (AACE) and American College of Endocrinology (ACE) Comprehensive Clinical Practice Guidelines for Medical Care of Patients with Obesity (AACE/ACE guidelines).^18^ Knowledge assessment attempted to review key areas comprising guideline‐directed obesity care, and excluded bariatric surgery treatments given their less likely presence in primary care environment. All questionnaire items can be found in Supporting Information S1.

A cohort of five primary care clinicians well‐versed in guideline‐directed obesity treatment served as expert reviewers to vet questionnaire items. Their involvement was aimed at ensuring instrument face validity, that the items met the study's goals, and that the intended data were accurately collected. Feedback on the instrument was collected via email correspondence and open‐ended questioning. Several questions were posed to clinicians for feedback. One question, for example, was “Do you feel the questions are relevant to the management of obesity in your practice?” Feedback responses were then collected to determine which components of the questionnaire appropriately evaluated clinician agreement with and knowledge of guideline‐directed obesity treatment. After review of feedback, slight adjustments to the questionnaire were made before finalization. Clinicians involved in corroborating survey questions did not participate in the study.

Survey responders were included if they were affiliated with the health system and provided patient care in a primary care location. Screening questions were utilized to ensure inclusion criteria were met for participating clinicians. Distribution of the questionnaire occurred electronically via email to 30 different Ascension Health entities across the United States (US). Ascension Health is comprised of hospitals and outpatient clinics that provide care in 19 states, including Kansas, and the District of Columbia.^19^ A call for participants was distributed in a regularly issued Ascension electronic newsletter and through directed Ascension leadership emails. Study investigators targeted leadership personnel to distribute the questionnaires that were determined to be closely connected to clinicians eligible for survey participation. Data collection occurred over a period of 34 days (28 February 2023 and 3 April 2023). REDCap^®^ software facilitated questionnaire distribution, completion, and data extraction.^20^, ^21^

Clinician agreement was measured on a 5‐point Likert scale, described by the strength of agreement or disagreement to each statement related to evidence‐based obesity treatment. Responses to clinician agreement questions were described further by range and median of answers. Knowledge assessment questions were measured as accurate or “correct” if the pre‐determined, evidence‐based answer was selected. Total scores were calculated for each survey respondent and as a whole out of a total of seven potentially correct answers. Imputation of missing data occurred. Missing data was analyzed as incorrect answers to knowledge assessment questions.

### 2.2 Retrospective patient data analysis

The study site for the secondary outcomes included a network of 12 primary care clinic locations within a single health system from Ascension Via Christi and Ascension Medical Group in Wichita, Kansas. The included clinic locations consisted of nine family medicine and internal medicine clinics and two family medicine residency clinics that are largely a teaching environment for medical residents. These clinic locations were limited to a single local health system due to time and resource constraints of the study investigators. The patient population was representative of the general population of the city of Wichita who were insured and cared for in the primary care setting due to the fact that Ascension Via Christi and Ascension Medical Group supply the majority of services related to this population. Patient data were collected retrospectively utilizing the electronic health record Cerner^®^ and scheduling platform SAP^®^ (PowerInsight). Patients were included if they had a documented diagnosis of obesity (International Classification of Disease 10 (ICD‐10) codes for obesity) or at least one documented body mass index (BMI) of ≥30 kg/m^2^ over a four‐year time period of 30 June 2016 and 30 June 2020.^22^ Patients were identified as having a weight‐related complication if at least one of the following were documented: dyslipidemia, hyperlipidemia, hypertension, knee osteoarthritis, or obstructive sleep apnea. Patients were excluded if they were pregnant, imprisoned, or diagnosed with one or more of the following conditions which have the potential to skew the accuracy of BMI interpretation: heart failure, ascites, amputations, or eating disorders. Imputation of missing data did not occur, and patients with incomplete data were included in the analysis for qualified variables based on available data.

### 2.3 Statistical analysis

Descriptive statistics were performed on data collected from the clinician survey and patient demographic data collected within the retrospective analysis. Associations between variables included Mann‐Whitney *U* test/independent samples *t*‐test for continuous variables, and likelihood ratio chi‐square for categorical variables. Statistical analysis was performed using SigmaPlot 14.5, Microsoft Excel^®^ software, IBM SPSS^®^ Statistics Version 26. Results were statistically significant if the *p* value of their analyses was below a predefined alpha level of 0.05.

## 3 RESULTS

### 3.1 Prospective clinician survey

A total of 30 clinicians participated in the survey. Three clinicians were ineligible based on inclusion criteria, and data from 27 participating clinicians were included in the analysis. There were 18 (67%) female participants, 19 (70%) physician participants, and 20 (74%) participants located in the state of Kansas. Participants were evenly distributed according to age and years in practice. See Table 1 for additional clinician demographic information.

**TABLE 1:** Demographic data of clinicians who participated in the prospective survey.

Survey participant results: Clinician demographics	
Characteristic	Participant count (*n* = 27)
Gender, No. (%)	
Female	18 (67)
State, No. (%)	
Kansas	20 (74.1)
Florida	2 (7.4)
Michigan	3 (11.1)
Tennessee	1 (3.7)
Texas	1 (3.7)
Occupation, No. (%)	
Non‐resident physician	13 (48.2)
Resident physician	6 (22.2)
Nurse practitioner	5 (18.5)
Physician associate	1 (3.7)
Other	1 (3.7)
Unspecified	1 (3.7)
Years in practice, No. (%)	
0–5	9 (33.4)
6–10	5 (18.5)
11–20	9 (33.3)
21–30	4 (14.8)
31 or more	0

**FIGURE 1:**
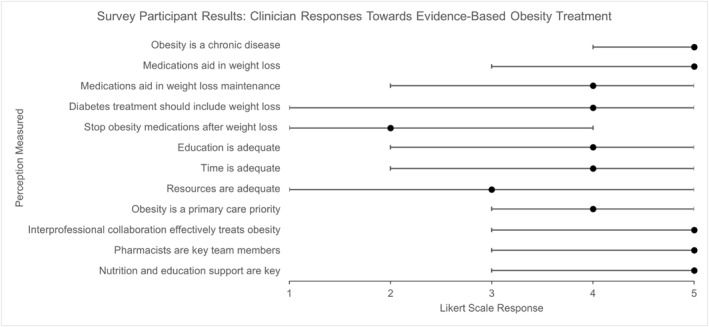
Survey Likert scale question responses depicting clinician perceptions. Likert scale: 1 = strongly disagree, 2 = disagree, 3 = neither agree nor disagree, 4 = agree, 5 = strongly agree.

Results of clinician agreement, including range and median for each questionnaire item are included in Figure 1. Subgroup analyses were performed to identify if statistically significant differences in agreement existed based on clinician occupation, years in practice, or age, and for knowledge scores based on clinician years in practice. No statistically significant associations were identified.

**FIGURE 2:**
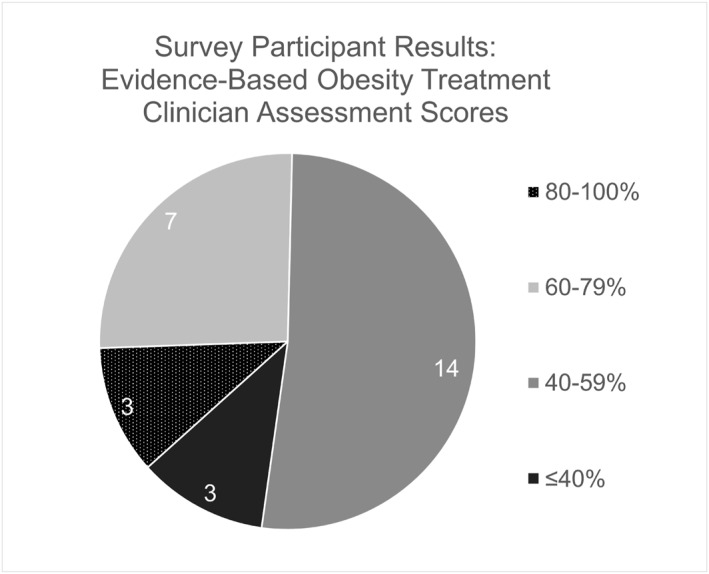
Survey data from clinician knowledge questions.

Knowledge of obesity treatment varied across domains assessed, with potential clinician gaps in knowledge identified. There were 14 (52%) participating clinicians that missed or did not respond to three to four out of the seven questions included. The number of missed questions ranged from one to six incorrect answers, with an average knowledge assessment score for all participants of 2.5/7 questions, representing an average score of 36% correct answers. A visual representation of these results is included in Figure 2. The most significant gaps in clinician knowledge identified related to selecting standard weight loss goals, with 14 (52%) clinicians selecting an incorrect response, baseline caloric deficit requirements for weight loss, with 16 (59%) selecting an incorrect response, and AOMs approved by the Food and Drug Administration (FDA), with 14 (52%) selecting an incorrect response.

Further analysis was conducted for 18 (67%) participants who reported stronger agreement (Likert scale responses of “agree” or “strongly agree”) toward adequate education of obesity in the primary care setting. These clinicians were marked as “confident” in evidence‐based obesity knowledge, and subsequently evaluated on whether they substantiated their confidence through their knowledge scores. Of the “confident” subset of clinicians, 10 (56%) missed more than two questions and achieved an assessment score of <60%.

### 3.2 Retrospective patient data analysis

A total of 14,544 clinic encounters from 5741 unique patients were identified during the collection period. Upon review of clinical data relating to BMI, concerns arose as to the accuracy of height and weight encounter data for patients whose calculated BMI fell below 18 kg/m^2^ or above 90 kg/m^2^. A total of 85 encounters (67 patients) were excluded based on BMIs calculated to be outside the range 18–90 kg/m^2^. A total of 657 baseline encounters and 212 most recent encounters had missing clinical data necessary for BMI calculation and were included in all analyses apart from those relating to BMI and obesity classification.

For patients with more than one encounter within the collection period, data from baseline and most recent encounters were evaluated. There were 5656 baseline encounters compared with 2941 most recent encounters. Mean patient age was 46 years. Across all encounters, most patients were female (>67%), had commercial insurance (>85%), and were treated by a family medicine clinician (>72%). See Table 2 for additional patient demographic data.

**TABLE 2:** Demographic data of patients analyzed in retrospective analysis.

Retrospective analysis results: Patient demographics		
Variable	Baseline encounter (*n* = 5656)	Most recent encounter (*n* = 2941)
Biological sex, no. (%)		
Female	3846 (68.0)	2022 (68.8)
Age		
Mean (SD), years	45.9 (12.4)	47.3 (12.1)
Median (IQR), years	47 (6.5)	48 (20.0)
Age group, no. (%), years		
18–33	1064 (18.8)	449 (15.2)
34–49	2172 (38.4)	1111 (37.8)
50–65	2420 (42.8)	1381 (47.0)
AOM prescribed, no. (%)		
Yes	1085 (19.2)	570 (19.4)
No	4571 (80.8)	2371 (80.6)
Weight‐related complication, no. (%)		
Yes	3209 (56.7)	1839 (62.5)
No	2447 (43.3)	1102 (37.2)
Insurance type, no. (%)		
Government	386 (6.8)	204 (6.9)
Commercial	4847 (85.7)	2529 (86.0)
Self‐pay/charity	119 (2.1)	57 (1.9)
Other	304 (5.4)	151 (5.1)
Encounter location, no. (%)		
Family medicine	4223 (74.7)	2128 (72.4)
Internal medicine	1433 (25.3)	813 (27.6)

Abbreviation: AOM, anti‐obesity medication.

**FIGURE 3:**
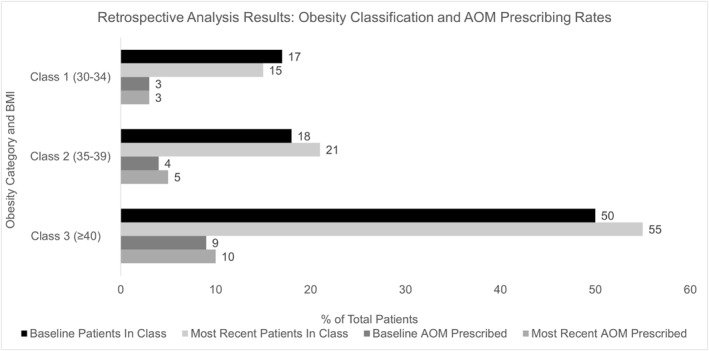
Clinical patient data from retrospective analysis. AOM, anti‐obesity medication, BMI, body mass index. *The increase in obesity severity over time (as shown by increasing rates of Class 2 and 3 obesity) was found to be statistically significant between baseline and most recent encounters.

Data revealed a comparable mean BMI between baseline and most recent encounters of 42 and 43 kg/m^2^, respectively. Across all encounters, a sizable portion of patients were classified in the most severe category of obesity (Class 3) with a BMI ≥40 kg/m^2^, with 2828 (50%) patients at baseline and 1629 (55%) patients at most recent encounters. Further confirming the severity of obesity in the study population was the presence of at least one weight‐related complication in 3209 (57%) patients at baseline and 1839 (63%) patients at most recent encounter. The number of patients prescribed AOMs at the time of their encounter were 1085 (19%) patients at baseline and 570 (19%) patients at the most recent encounter. See Figure 3 for a visual representation of both obesity classification and AOM prescribing results. Statistically significant differences were seen in patient age, obesity class, type of AOM prescribed, and treating clinician specialty between baseline and most recent encounters.

**FIGURE 4:**
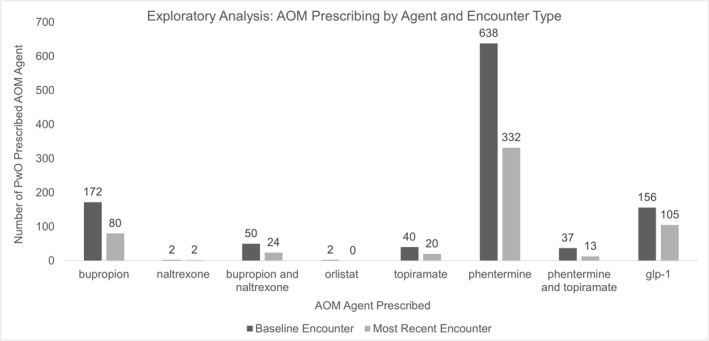
Medication prescribing details for patients analyzed in retrospective analysis. AOM, anti‐obesity medication. *Differences in AOM agent prescribed were found to be statistically significant between baseline and most recent encounters.

An exploratory analysis of patient AOM data was conducted. Phentermine monotherapy was the most common AOM prescribed, including 638 (59%) patients at baseline and 332 (59%) patients at most recent encounter. Agents within the glucagon like peptide‐1 (GLP‐1) treatment class were least commonly prescribed, including 156 (14%) patients at baseline and 105 (18%) patients at most recent encounter. See Figure 4 for additional results of the type of AOM prescribed across all encounters.

## 4 DISCUSSION

Clinicians reported stronger agreement toward the consideration of obesity as a chronic disease, and toward medication use to achieve weight loss. Clinicians also reported stronger agreement toward prioritizing obesity treatment in the primary care setting and utilizing interprofessional collaboration in obesity treatment. We believe that the strong agreement identified in this study represents positive clinician perceptions toward obesity care, is encouraging with regard to reducing stigma, and may support prioritizing interprofessional collaboration within the primary care setting as a way to deliver care for persons with obesity. Integration of clinicians such as exercise specialists, certified diabetes educators, and behavioral health specialists may be well‐received and utilized by existing primary care clinicians.

There was less agreement toward the chronic use of AOMs to maintain weight loss. Varied responses existed toward adequate education and time for obesity treatment in the primary care setting. Disagreement was strongest regarding resource availability for obesity treatment in primary care. This may represent negative perceptions of clinicians toward chronic AOM use, which invites discussion of the importance of clinicians utilizing evidence‐based obesity practices for this intervention type. An increase in clinicians prioritizing evidence‐based practices may lead to increased patient engagement with obesity treatment. This concept is supported by data from a 2018 study by Bloom et al. where patients described positive perceptions toward the use of evidence‐based obesity treatment.^23^

Both gaps in knowledge and a lack of congruence between perceived and actual knowledge of clinicians were identified. Of the total incorrect responses recorded (79 incorrect, 3 missing), 23 responses (28%) were related to appropriate medication use in obesity treatment and 24 (29%) were related to the pathophysiology of obesity. Collaborating clinicians, such as pharmacists and dieticians, may be uniquely situated to help close these knowledge gaps as part of an interprofessional team given their unique skill sets and viewpoints directly related to these knowledge gaps.

Questionnaire items utilized in this study focused on agreement with evidence‐based medication use. A limitation of this study is that items evaluating prescribing practices of AOMs were not included. Given this limitation, it is unknown whether clinicians participating in this study use prescribing practices in alignment with their beliefs on AOM use. In a similarly conducted 2018 study by Falvo et al., investigators found that participating primary care clinicians rarely prescribed medications for obesity.^17^

The limited sample size of this survey significantly impacts the applicability of its results to the general population of primary care clinicians. Additionally, the limited geographic practice location of participants in the Midwest as well as the use of a survey instrument which was not validated externally impact the results of our study. Possible contributing factors toward a low sample size include the method of questionnaire distribution, limited timeline response, and challenges inherent to the nature of survey‐based research. However, helpful insights can be derived from survey respondent data to describe obesity treatment in this practice setting.

An additional limitation is the reporting of missing data for clinician knowledge assessment questions as incorrect, potentially skewing data toward larger provider knowledge gaps, although missing data represented a very small portion of answers. Components related to how survey responders could report lack of knowledge or lack of willingness to participate in specific questionnaire items were not included in the survey instrument, limiting the instrument's strength and accuracy of data interpretation. The survey instrument also did not allow clinicians to specify reasons as to their agreement or disagreement. This information, particularly surrounding specific resource need(s), is important in targeting effective means of improving resource availability. Although not evaluated, a connection may exist between what we believe to be negative perceptions on resource availability and support for interprofessional care reported by clinicians in this survey. Interprofessional care may help alleviate projected clinician shortages in the primary care setting and aid in resource availability related to obesity.

Analysis took place with patient data from a single health system located in the city of Wichita, Kansas, which may limit the applicability of its results outside of this region. In 2016, the population of adult persons with obesity was estimated to be >30% in Kansas, which grew to >35% by 2020.^24^, ^25^ The city of Wichita has experienced a steady rise in the rates of persons with obesity reaching an estimated prevalence of 40% in 2021, comparable to that of the national average at the time of data collection.^25^, ^26^ This study is useful in helping clinicians better understand the current landscape of treatment of persons with obesity and the changes which must be made to address the highly prevalent need for obesity treatment in this city and state.

Many patients analyzed in this study experienced a notable severity of obesity, shown by a high prevalence of patients with BMI ≥40 kg/m^2^ and one or more weight‐related complications. These results are likely an accurate reflection of the patient population across the study's chosen health‐system, as all primary care clinics were included in the data analysis.

AOMs vary significantly in their ability to produce clinically meaningful sustained weight loss. The use of phentermine monotherapy in obesity treatment is not recommended per AACE/ACE guidelines and is less strongly recommended in other guidelines on obesity and diabetes treatment.^18^, ^27^, ^28^, ^29^, ^30^, ^31^ While phentermine in combination with topiramate is an AOM approved for chronic use, phentermine monotherapy has only gained approval for short‐term use (≤3 months) based on safety and efficacy data.^32^ Based on this study's exploratory analysis results, it appears that the highly prevalent yet seemingly undesirable practice of phentermine monotherapy persists, as this practice occurred in 59% and 62% of total AOM prescriptions at baseline and most recent encounters, respectively. However, consensus on the acceptability of phentermine monotherapy may be shifting as the appeal for affordable AOMs grows in the US healthcare market.^33^

Unlike phentermine monotherapy, use of GLP‐1s as monotherapy is strongly recommended by newer obesity and diabetes management guidelines, including AACE/ACE guidelines, given the robust data supporting their efficacy as an AOM.^18^, ^27^, ^28^, ^29^, ^30^, ^31^ In this study, prescribing within the GLP‐1 class was limited when compared to other AOM classes, shown by low prescribing rates of 14% and 18% of total AOM prescriptions at baseline and most recent encounters, respectively. Data were collected across a span of time (2016–2020) during which implementation AACE/ACE guidelines on obesity treatment would be expected. It is unclear why implementation of these guidelines was lacking, although potential factors include clinician resistance and limited resource availability. If data were to be collected in more recent years (i.e., 2020–2024), an increase in GLP‐1 prescribing may have been shown given their growing popularity and recommended use for obesity treatment.^30^, ^33^, ^34^

All GLP‐1s are contraindicated in patients with a personal or family history of medullary thyroid cancer and are either contraindicated or cautioned in pancreatitis.^35^, ^36^, ^37^, ^38^, ^39^ Due to their mechanism of action of slowing gastric emptying, many clinicians also avoid the use of GLP‐1s in patients with gastroparesis. In this study, patient encounters which had one or more documented ICD‐10 codes of any thyroid cancer, pancreatitis, or gastroparesis were considered ineligible for GLP‐1 therapy. Based on these criteria, data from 66 encounters excluded patients from GLP‐1 therapy, leaving the remaining 14,478 encounters as potential opportunities for GLP‐1 prescribing.

Both the high use of phentermine monotherapy and the gap in prescribing GLP‐1s identified in this study are concerning. A potential reason for the gap in GLP‐1 prescribing could be their high cost, coupled with the common barrier of lack of AOM coverage by prescription insurance plans. However, one year prior to the start of the data collection period, a source indicated that approximately two thirds of all insurance companies offered coverage for at least one form of AOM use.^40^ Still, the high use of phentermine monotherapy may support the conclusion that AOM coverage was lacking given its low cost compared to other AOMs. Coverage for GLP‐1s indicated for obesity treatment has been met with challenges, escalating to legislative efforts to overcome these challenges.^41^

The prospective survey conducted in this study did not align with the time of retrospective patient data collection, limiting the association between survey results and patient data. However, consistently concerning results were shown in both methods with regard to the chronic use of AOMs, which may suggest that future research is needed to describe the relationship between perceptions of chronic AOM use and clinician prescribing patterns.

A limitation of this retrospective analysis is the lack of control over documented data quality, specifically for ICD codes and height and weight data for BMI calculations. The formal documentation of obesity as a disease using well‐known systems such as ICD codes is important as past data have shown this documentation strongly influences treatment of this disease.^42^ Medication indications were not collected, which could impact the accuracy of AOM data. Currently, two FDA combination oral therapies are approved AOMs. Monotherapy use of agents within these combinations is appropriate for non‐obese indications. This study was not able to ascertain and exclude appropriate monotherapy use of single agents approved in an FDA approved combination AOM. The retrospective analysis did not include a significant portion of patient data collected during the time of the 2019 Novel Coronavirus public health emergency, which began in January of 2020 and was later declared a pandemic in March of 2020.^43^ The study's scope lacked the ability to effectively control for confounding factors resulting from the pandemic such as reduced access to obesity treatment services.

The analysis did not include other key factors used to assess guideline‐directed care, such as the pattern of follow‐up appointments addressing obesity, trials and intolerances of AOMs, adherence to prescribed AOMs, and concomitant lifestyle interventions. This study also did not report rates of specific weight‐related complications that were screened for, nor did it screen for the comprehensive range of weight‐related complications that exist. The results from this study are idea‐generating for future research into how guideline‐directed obesity treatment can be realized in the primary care setting. Quality improvement initiatives in the form of education may be beneficial for primary care clinicians. Interventions and advocacy for improved coverage of AOMs or implementation of clinical pharmacist collaboration may prove beneficial for both clinicians and persons with obesity helping meet the need that both have in achieving optimal obesity care.

## 5 CONCLUSION

This study demonstrated that primary care clinicians are in general in agreement with evidence‐based obesity treatment and have needs in applying knowledge of guideline‐directed practices and obtaining resources to care for persons with obesity. Clinical markers of high BMI and weight‐related complications for persons with obesity identified in this study appear to warrant significant improvement in the management of persons with obesity, particularly in the use of AOMs.

## CONFLICT OF INTEREST STATEMENT

None to disclose for all authors.

## Supporting information

Supporting Information S1
