# Supplementary material for: Assessment of clinician agreement with and knowledge of evidence‐based obesity treatment in the primary care setting
Source: Obes Sci Pract. 2024 Oct 1;10(5):e70011. doi: 10.1002/osp4.70011 (PMC11443603; doi:10.1002/osp4.70011)
Supplement: Supplementary file 1 — Supporting Information S1 [file OSP4-10-e70011-s001.docx]

**Appendix A: Survey Questionnaire Items**

Questions 1-12 below utilized a 5-point Likert scale (1 = strongly disagree, 2 = disagree, 3 = neither agree or disagree, 4 = agree, 5 = strongly agree):

1. Evidence supports that obesity is a chronic disease requiring lifelong management.
2. There are medications which help patients to effectively achieve weight loss.
3. There are medications which help patients to effectively maintain weight loss.
4. Treatment strategies for patients who have obesity and diabetes should prioritize weight loss.
5. Pharmacologic treatments for obesity should be discontinued once the patient has met their recommended weight loss goal.
6. I have adequate education regarding effective obesity treatment to meet patient needs.
7. I have adequate time for effective obesity treatment to meet patient needs.
8. I have adequate resources for effective obesity treatment to meet patient needs.
9. Treatment of obesity is a key responsibility of primary care clinicians.
10. An interprofessional approach is needed to effectively treat obesity in the primary care setting.
11. Clinical pharmacists (PharmDs) functioning in primary care settings are key collaborators in effective obesity treatment.
12. Dieticians (RDs), certified diabetes educators, and nutritionists functioning in primary care settings are key collaborators in effective obesity treatment.

Questions 13-19 below were multiple choice (correct answers are marked with an *):

1. The metabolic adaptations which occur in obesity involve which of the following hormones?
   1. Leptin (satiety hormone)*
   2. Ghrelin (hunger hormone)*
   3. Somatotropin (growth hormone)
   4. Adrenaline
   5. Prolactin
2. Which of the following are well-known, established weight-related complications?
   1. Type 2 diabetes mellitus*
   2. Anemia
   3. Dyslipidemia*
   4. Polycystic ovarian syndrome*
   5. Osteoporosis
3. What is the standard initial weight loss goal recommended for obesity treatment?
   1. 3%
   2. 5%*
   3. 8%
   4. 10%
   5. 15%
4. Which of the following medications are FDA approved for obesity treatment?
   1. Semaglutide*
   2. Metformin
   3. Bupropion
   4. Orlistat*
5. Which of the following therapies have a reasonable risk for causing weight gain?
   1. Topiramate
   2. Mirtazapine*
   3. Insulin*
   4. Atorvastatin
   5. Quetiapine*
6. Which is a preferred form of behavioral modification for obesity treatment?
   1. Negative reinforcement
   2. Increasing stimulus exposure
   3. Virtual, one-on-one meetings
   4. Clear, reasonable goal-setting*
7. What is the recommended kcal per day deficit recommended for patients with obesity attempting to achieve weight loss?
   1. 100-250
   2. 251-499
   3. 500-750*
   4. 751-999
